# Unit Root Testing and Estimation in Nonlinear ESTAR Models with Normal and Non-Normal Errors

**DOI:** 10.1371/journal.pone.0166990

**Published:** 2016-11-29

**Authors:** Umair Khalil, Alamgir, Amjad Ali, Dost Muhammad Khan, Sajjad Ahmad Khan, Zardad Khan

**Affiliations:** 1 Department of Statistics, Abdul wali Khan University Mardan, Mardan, Pakistan; 2 Department of Statistics, University of Peshawar, Peshawar, Pakistan; 3 Department of Statistics, Islamia College Peshawar, Peshawar, Pakistan; Universidad de Granada, SPAIN

## Abstract

Exponential Smooth Transition Autoregressive (ESTAR) models can capture non-linear adjustment of the deviations from equilibrium conditions which may explain the economic behavior of many variables that appear non stationary from a linear viewpoint. Many researchers employ the Kapetanios test which has a unit root as the null and a stationary nonlinear model as the alternative. However this test statistics is based on the assumption of normally distributed errors in the DGP. Cook has analyzed the size of the nonlinear unit root of this test in the presence of heavy-tailed innovation process and obtained the critical values for both finite variance and infinite variance cases. However the test statistics of Cook are oversized. It has been found by researchers that using conventional tests is dangerous though the best performance among these is a HCCME. The over sizing for LM tests can be reduced by employing fixed design wild bootstrap remedies which provide a valuable alternative to the conventional tests. In this paper the size of the Kapetanios test statistic employing hetroscedastic consistent covariance matrices has been derived and the results are reported for various sample sizes in which size distortion is reduced. The properties for estimates of ESTAR models have been investigated when errors are assumed non-normal. We compare the results obtained through the fitting of nonlinear least square with that of the quantile regression fitting in the presence of outliers and the error distribution was considered to be from t-distribution for various sample sizes.

## Introduction

The theoretical perception that transactions costs can bring non-linear change of deviation from equilibrium circumstances ([1], [2], [3], [4], [5]) can describe the economic performance from a linear perspective of various variable that look non-stationary. One nonlinear form that captures this type of behavior is the smooth transition model ESTAR model. Nonlinear models of the ESTAR form can imply near unit root behavior near equilibrium. These models have been a great choice for nonlinear modeling in non-stationary context.

A few researchers ([6], [7], [8] and [9]) also worked on smooth transition model. Kapetanios G., et al. [10], worked on the unit root testing of particular kind in nonlinear dynamics and provides an alternative frame work to test the unit root against nonlinear ESTAR, which is globally stationary.

Kapetanios G., et al. [10] has suggested a univariate testing procedure to identify the existence of nonstationarity against the nonlinear ESTAR process. He has also derived a nonstandard limiting distribution of Kapetanios tests. He then has examined the power/size performance of the small sample size and has found that in many cases under the alternative stationary ESTAR process has a power gain over the Dickey fuller test. We are going to use this test but with different error distribution to find the critical values and then the size and power of the test. Cook [11] states that when the degrees of freedom of the t-distribution innovations are lower, the amount of oversizing is larger. Due to that reason we use 5 degrees of freedom in the innovation process. Cook has found the results that experts may experience false rejection while studying heavy tailed data when they use normally distributed errors for obtaining critical values.

More or less every function which is included in nonlinear regression model can be written in closed form. The use of parameters in the functional part of a nonlinear regression model is rarely studied. We have used the quantile fitting and had compared it with the nonlinear least square for the ESTAR model in the presence of outliers. This technique proposes a system for estimating models for the conditional quantile function and the median function. To study the stochastic relationships among random variables, quantile regression is capable of providing a more complete statistical analysis.

## Smooth Transaction Autoregressive Models

The smooth transition autoregressive (STAR) model for a univariate time series *y*_*t*_, at time *t* = 1−*p*,1−(*p*−1),…………,−1,0,1,….*T*−1,*T* is given by

$$\begin{array}{c} y_{t}=(\phi_{1,0}+\phi_{1,1}y_{t-1}+\cdots +\phi_{1,p}y_{t-p})(1-F(s_{t};\gamma ,c))+(\phi_{2,0}+\phi_{2,1}y_{t-1}+\cdots +\phi_{2,p}y_{t-p})F(s_{t};\gamma ,c)+\varepsilon_{t}\\ t=1,\ldots \ldots \ldots ..,T \end{array}$$ (1)

The *ε*_*t*_’_s_ are assumed to have 0 mean and a constant variance equals to *σ*^2^. The transition function *F*(*s*_*t*_;*γ*,*c*) is a continuous function bounded between 0 to 1. It was discussed [12] that the transition variable *s*_*t*_ = *y*_*t*−*d*_ for certain integer *d* > 0 in the STAR model is assumed to be a lagged endogenous variable. Shin et al. [13] also worked on STAR model.

The structure of the model at time *t* depends on the variable *s*_*t*_ and the value of *F*(*s*_*t*_;*γ*,*c*). The choice of *F*(*s*_*t*_;*γ*,*c*) gives birth to different categories of models. This includes logistic STAR (LSTAR) model, self-exciting TAR (SETAR) model used by [14] and [15] in experimental studies.

In many cases when the domains are associated with small and large absolute values of *s*_*t*_, it is more suitable to specify the transition function. For this purpose the exponential type function can be used such as

$$F(\theta ;s_{t},c)=1-\exp[-\theta {\left(s_{t}-c\right)}^{2}],\;\gamma >0$$ (2)

Where *γ* evaluate the tempo of shift from one domain to another and signifies the location for threshold value for *s*_*t*_. Replacing for the exponential function of Eq (2) in Eq (1) we get the exponential smooth transition autoregressive model. The transition function of ESTAR is symmetric and U-shaped around *c*. Michael et al. (1997), Sarantis (1999) and Taylor et al. (2001) applied this model has been applied to real exchange rate by a few researchers ([16], [17]).

## Testing Unit Root Hypothesis against ESTAR Using Heteroscedastic Consistent Covariance Matrix Estimators (HCCM)

We will consider the following univariate ESTAR model of order 1

$$y_{t}=\phi y_{t-1}+\gamma y_{t-1}[1-\exp\{-\theta {\left(s_{t}-c\right)}^{2}\}]+\varepsilon_{t}$$ (3)

the model used by [10]. Rearranging Eq (3) putting *c* = 0 and replacing the transition variable *s*_*t*_ by a lagged dependent variable, *y*_*t*−*d*_ for *d* > 0 gives us

$$\Delta y_{t}=\beta y_{t-1}+\gamma y_{t-1}[1-\exp\{-\theta y_{t-d}^{2}\}]+\varepsilon_{t}$$ (4)

Where *β* = *ϕ*−1. Using the conditions *β* ≥ 0, *γ* < 0 and *β* + *γ* < 0, Kapetanios has proved the geometric ergodicity for the ESTAR process.

Putting *β* = 0, in Eq (4) consequently giving that in the central regime *y*_*t*_ trail a unit root process. We set the delay parameter *d =* 1, which gives

$$\Delta y_{t}=\gamma y_{t-1}[1-\exp\{-\theta y_{t-1}^{2}\}]+\varepsilon_{t}$$ (5)

Our null hypothesis here will be that H_0_: *θ* = 0 against the alternative hypothesis that H_1:_ *θ* > 0. Testing the above null hypothesis is not practicable because of the fact that under the null hypothesis *γ* is not recognized. The issue has been resolved in [10] by introducing a *t*-type test using the auxiliary regression

$$\Delta y_{t}=\delta y_{t-1}^{3}+error$$ (6)

This has been computed from the first-order Taylor approximation to ESTAR. The Kapetanios *t*-statistics suggested is

$$t_{NL}=\hat{\delta }/s.e(\hat{\delta })$$ (7)

Where $\hat{\delta }$ is the estimate of *δ* and $s.e(\hat{\delta })$ is the standard error of $\hat{\delta }$. Table 1 bellow shows the appropriate asymptotic critical values calculated from the simulations studies with DGP used as AR(1) process with unit root hypothesis

$$y_{t}=y_{t-1}+\varepsilon_{t}$$ (8)

**Table 1:** Asymptotic critical values for the Kapetanios t-statistics with sample size equals 10,000.

Error distn.	Random normal			Random t-disttn.			Gamma		
Fractile	1%	5%	10%	1%	5%	10%	1%	5%	10%
Case 1	-2.804	-2.223	-1.926	-2.841	-2.217	-1.924	-3.815	-3.454	-3.230
Case 2	-2.872	-2.258	-1.953	-2.817	-2.219	-1.929	-6.385	-5.162	-4.487
Case 3	-2.860	-2.248	-1.945	-2.805	-2.209	-1.921	-5.953	-4.675	-4.139
Case 4	-2.847	-2.239	-1.937	-2.792	-2.201	-1.914	-5.560	-4.380	-3.830
Error distn.	Cauchy			Poisson (*λ* = 4).			Log-normal		
Fractile	1%	5%	10%	1%	5%	10%	1%	5%	10%
Case 1	-3.534	-2.158	-1.637	-3.815	-3.454	-3.230	-10.443	-11.919	-12.492
Case 2	-2.140	-1.702	-1.479	-6.385	-5.162	-4.487	-9.938	-12.196	-13.273
Case 3	-2.134	-1.696	-1.473	-5.953	-4.675	-4.139	-9.913	-12.170	-13.246
Case 4	-2.130	-1.690	-1.469	-5.560	-4.380	-3.830	-9.896	-12.142	-13.217

With sample size 10,000 and with 50,000 replications. In the general case (case 1) we use the constant variance (homoscedasticity) in the estimation of the asymptotic critical values. Using conventional tests is dangerous [18] though the best performance among these is a HCCME used by [19]. We have also used the hetroscedastic consistent covariance matrix (HCCM) in the estimation of standard error which was introduced by [20] and [21]. In case 2 we have used the HC0, which estimates the provisional variance of the error for each mold of *y*_*i*_. HC0 is defined as

$$HC0={\left(Y^{\prime }Y\right)}^{-1}Y^{\prime }diag({\hat{e}}_{t}^{2})Y{\left(Y^{\prime }Y\right)}^{-1}$$ (9)

In the presence of an unknown form, HC0 is a consistent estimator of the variance of the parameter.

In case 3 and 4 we have used the HC2 and HC3 which adjusts each $\varepsilon_{i}^{2}$ as how much the case influences the estimates of the coefficient. Mackinnon and White [19] proposed

$$HC2={\left(Y^{\prime }Y\right)}^{-1}Y^{\prime }diag(\frac{{\hat{e}}_{t}^{2}}{1-h_{i}})Y{\left(Y^{\prime }Y\right)}^{-1}$$ (10)

Where *h*_*i*_ is the *i*^*th*^ element of the projection matrix. If the model is homoscedastic in actual fact, this estimate is unbiased. A third variation approximates additional intricate Jacknife estimator of Efron [22] as

$$HC3={\left(Y^{\prime }Y\right)}^{-1}Y^{\prime }diag(\frac{{\hat{e}}_{t}^{2}}{{\left(1-h_{i}\right)}^{2}})Y{\left(Y^{\prime }Y\right)}^{-1}$$ (11)

HC3 constructs confidence intervals that lean to be yet further conventional. HC0 was outperformed by HC2 and HC3

## Using Wild Bootstrap Technique to Find the Size of Test in ESTAR Models

The bootstrap is a computationally rigorous technique commenced by [23]. Bootstrap resampling methods have been come forward as an authoritative tool for building inferential procedures in contemporary statistical data analysis. The inferential constituent of a statistical analysis typically consists of constructing confidence intervals, connecting a standard error to an estimator, hypothesis testing, prediction region constructing or choosing a regression equation. Habitually it is wholly required to estimate the sampling distribution of some statistic. There are different types of bootstrap resampling. We are using the wild bootstrap technique for finding the size of our test which has improvement over other resampling in small sample cases. It has been shown [24] that using Wild Bootstrap where there is no hetroscedasticity gives "better results" than employing standard bootstrap (with replacement) when there is hetroscedasticity. In this technique we resample with replacement the residuals that have been estimated from the initial fit, multiplied randomly by -1 or 1.Symmetry is assumed in this technique for the true residual distribution. To find the size of the test we use the DGP in Eq (8) with *ε*_*t*_ drawn from t-distribution with 5 degrees of freedom and standard normal distribution separately in two tables below. The results shown in Table 2 and Table 3 using Kapetanios t-statistics and using wild-bootstrap technique respectively with 50,000 replications and the bootstrap sample has been taken to be 1000.

**Table 2:** The size of the test for Kapetanios t-statistics.

Error disttn.	Random normal			Random t-disttn.		
Fractile	1%	5%	10%	1%	5%	10%
N = 50	0.011	0.046	0.930	0.013	0.045	0.085
N = 100	0.015	0.047	0.101	0.007	0.049	0.092
N = 250	0.011	0.055	0.114	0.010	0.054	0.096
N = 500	0.019	0.059	0.109	0.010	0.045	0.091
N = 1000	0.013	0.053	0.104	0.008	0.047	0.103

**Table 3:** The size of the test using wild bootstrap procedure.

Error disttn.	Random normal			Random t-disttn.		
Fractile	1%	5%	10%	1%	5%	10%
N = 50	0.010	0.051	0.110	0.015	0.061	0.103
N = 100	0.011	0.051	0.086	0.011	0.051	0.096
N = 250	0.017	0.056	0.108	0.011	0.054	0.110
N = 500	0.013	0.040	0.870	0.007	0.043	0.093
N = 1000	0.015	0.054	0.100	0.013	0.054	0.085

It is evident from the above table that the size of the test improves for Kapetanios test using the wild bootstrap technique.

## Comparing Power of Kapetanios Test with Wild Bootstrap Procedure

For the comparison of the power performance of the two methods under discussion we use *γ* = −1, *θ* = 0.05, 0.02 and 0.001 in the data generation process

$$\Delta y_{t}=\gamma y_{t-1}[1-\exp\{-\theta y_{t-1}^{2}\}]+\varepsilon_{t}$$ (12)

And the error distribution in the DGP is taken from random normal and random t-distribution with 5 degrees of freedom, which is the finite variance case so that the variance, skewness and shape of the distribution can be found if needed. Results are shown in the following Tables 4–9.

**Table 4:** The power of the test using Kapetanios t-statistics with theta = 0.05.

Error disttn.	Random normal			Random t-disttn.		
Fractile	1%	5%	10%	1%	5%	10%
N = 50	0.171	0.361	0.676	0.401	0.691	0.741
N = 100	0.611	0.744	0.799	0.896	0.899	0.910
N = 250	0.906	0.965	0.967	0.923	0.969	0.955
N = 500	0.971	0.977	0.984	0.974	0.987	0.990
N = 1000	0.999	1.00	1.00	0.988	0.998	1.00

**Table 5:** The power of the test for Kapetanios t-statistics with theta = 0.02.

Error distn.	Random normal			Random t-disttn.		
Fractile	1%	5%	10%	1%	5%	10%
N = 50	0.195	0.550	0.665	0.412	0.724	0.784
N = 100	0.771	0.790	0.805	0.906	0.911	0.939
N = 250	0.919	0.932	0.955	0.943	0.957	0.970
N = 500	0.991	0.994	0.999	0.980	0.989	0.994
N = 1000	1.00	1.00	1.00	1.00	1.00	1.00

**Table 6:** The power of the test using Kapetanios t-statistics with theta = 0.001.

Error distn.	Random normal			Random t-disttn.		
Fractile	1%	5%	10%	1%	5%	10%
N = 50	0.361	0.672	0.811	0.761	0.866	0.941
N = 100	0.819	0.881	0.915	0.913	0.961	0.980
N = 250	0.896	0.953	0.994	0.974	0.979	0.991
N = 500	0.990	0.996	1.00	0.999	1.00	1.00
N = 1000	1.00	1.00	1.00	1.00	1.00	1.00

**Table 7:** The power of the test using wild bootstrap procedure with theta = 0.05.

Error disttn.	Random normal			Random t-disttn.		
Fractile	1%	5%	10%	1%	5%	10%
N = 50	0.348	0.578	0.695	0.202	0.365	0.466
N = 100	0.567	0.766	0.844	0.254	0.398	0.483
N = 250	0.855	0.899	0.912	0.369	0.417	0.479
N = 500	0.925	0.941	0.946	0.541	0.719	0.768
N = 1000	0.933	0.952	0.992	0.607	0.727	0.802

**Table 8:** The power of the test using wild bootstrap procedure with theta = 0.02.

Error disttn.	Random normal			Random t-disttn.		
Fractile	1%	5%	10%	1%	5%	10%
N = 50	0.450	0.686	0.809	0.336	0.553	0.658
N = 100	0.754	0.904	0.951	0.469	0.628	0.716
N = 250	0.981	0.996	0.999	0.587	0.678	0.737
N = 500	0.998	1.00	1.00	0.641	0.719	0.768
N = 1000	1.00	1.00	1.00	0.659	0.727	0.779

**Table 9:** The power of the test using wild bootstrap procedure with theta = 0.001.

Error disttn.	Random normal			Random t-disttn.		
Fractile	1%	5%	10%	1%	5%	10%
N = 50	0.575	0.851	0.925	0.530	0.814	0.900
N = 100	0.932	0.988	0.995	0.818	0.931	0.966
N = 250	0.999	1.00	1.00	0.960	0.978	0.985
N = 500	1.00	1.00	1.00	0.978	0.986	0.990
N = 1000	1.00	1.00	1.00	0.982	0.989	0.990

Outliers are generated on the same process of contamination which is discussed below in section 6 to check as if it effect the power of the Kapetanios test or not. Tables 4 to 6 show that in case of contaminated data there is a significant decrease in the power of the Kapetanios test.

The above tables show that results in comparing the power performance of the two methods. It results in the significance increase in the power using the bootstrap technique when the errors are generated from normal distribution. In case when errors are generated from t-distribution, Kapetanios test performs better. Extreme care should be taken in applying the resampling technique when there are outliers especially when taking large samples from such data may give very misleading results.

## Comparing the Power of Nonlinear Least Square with Quantile Regression Fit in the Presence of Outliers

Quantile regression has been introduced by [25], is a statistical method planned to estimate, and carry out inference about, restricted quantile functions. Quantile regression technique proposes a system for estimating models for the provisional median function, and the complete range of other conditional quantile functions. This function is able to provide a more precise analysis of the associations between random variables by combining the estimation of restricted mean functions and procedure for estimating a full class of restricted quantile functions.

The DGP used for the purpose of comparison between the quantile fit and the least square regression in the nonlinear models is as follows

$$y_{t}=y_{t-1}[\exp\{-\theta {\left(y_{t-1}\right)}^{2}\}]+\varepsilon_{t}$$ (13)

Where *ε*_*t*_ is normally distributed with zero mean and a constant variance equals to 1 for Tables 10 and 11 and *ε*_*t*_ has a t-distribution with d.f equals 5 in Tables 12, 13 and 14.

**Table 10:** Power of test for nonlinear least square and nonlinear quantile fitting using error disttn as normal, with a’s the Coefficients of nonlinear model and b’s are the coefficients of quantile fit, with sample size equals 100.

	theta = 5 (a1 = -0.01a2 = -0.1)	theta = 5(a1 = -0.01a2 = -0.001)	theta = 5(a1 = 0a2 = -0.01)	theta = 5(a1 = 0a2 = -0.001)	theta = 15(a1 = 0a2 = -0.1)	theta = 15(a1 = -0.01a2 = -0.2)	theta = 15(a1 = -0.01a2 = -0.3)	theta = 15(a1 = 0a2 = -0.01)
a_1_	0.909	0.999	0.998	0.995	0.999	1.00	0.998	1.00
b_1_	0.860	0.921	0.939	0.900	0.980	0.897	0.913	0.998
**a2**	**0.873**	**0.913**	**0.900**	**0.968**	**0.931**	**0.943**	**0.966**	**0.981**
**b2**	**0.905**	**0.985**	**0.961**	**0.980**	**0.994**	**0.990**	**0.972**	**0.989**
a_3_	0.99	1.00	0.999	1.00	0.993	0.998	1.00	0.976
b_3_	1.00	1.00	1.00	1.00	1.00	1.00	1.00	1.00

**Table 11:** Power of test for nonlinear least square and nonlinear quantile fitting using error disttn as normal with a’s the Coefficients of nonlinear model and b’s are the coefficients of quantile fit, with sample size equals 250.

	theta = 5(a1 = -0.01a2 = -0.1)	theta = 5(a1 = -0.01a2 = -0.001)	theta = 5(a1 = 0a2 = -0.01)	theta = 5(a1 = 0a2 = -0.001)	theta = 15(a1 = 0a2 = -0.1)	theta = 15(a1 = -0.01a2 = -0.2)	theta = 15(a1 = -0.01a2 = -0.3)	theta = 15(a1 = 0a2 = -0.01)
a_1_	0.990	1.00	0.999	0.998	1.00	1.00	1.00	1.00
b_1_	0.981	0.970	0.985	0.983	0.992	0.987	1.00	0.999
**a2**	**1.00**	**0.982**	**0.998**	**0.990**	**0.980**	**0.993**	**0.991**	**0.899**
**b2**	**1.00**	**0.987**	**1.00**	**0.999**	**1.00**	**0.999**	**1.00**	**0.990**
a_3_	1.00	1.00	1.00	1.00	1.00	0.998	0.999	0.999
b_3_	1.00	1.00	1.00	1.00	1.00	1.00	1.00	1.00

**Table 12:** Power of test for nonlinear least square and nonlinear quantile fitting using error disttn as t(5) with a’s the coefficients of nonlinear model and b’s are the coefficients of quantile fit, with sample size equals 100.

	theta = 5(a1 = -0.01a2 = -0.1)	theta = 5(a1 = -0.01a2 = -0.001)	theta = 5(a1 = 0a2 = -0.01)	theta = 5(a1 = 0a2 = -0.001)	theta = 15(a1 = 0a2 = -0.1)	theta = 15(a1 = -0.01a2 = -0.2)	theta = 15(a1 = -0.01a2 = -0.3)	theta = 15(a1 = 0a2 = -0.01)
a_1_	0.978	0.980	1.00	0.999	0.991	0.992	0.999	0.995
b_1_	0.926	0.900	0.912	0.892	0.995	0.882	0.901	0.990
**a2**	**0.843**	**0.891**	**0.99**	**0.889**	**0.913**	**0.923**	**0.876**	**0.901**
**b2**	**0.920**	**0.976**	**0.966**	**0.912**	**0.995**	**0.999**	**0.923**	**0.998**
a_3_	0.965	0.971	0.982	0.991	0.975	0.991	0.940	0.932
b_3_	0.972	0.983	0.990	0.994	1.00	1.00	0.970	0.969

**Table 13:** Power of test for nonlinear least square and nonlinear quantile fitting using error disttn as t(5) with a’s the coefficients of nonlinear model and b’s are the coefficients of quantile fit, with sample size equals 250.

	theta = 5(a1 = -0.01a2 = -0.1)	theta = 5(a1 = -0.01a2 = -0.001)	theta = 5(a1 = 0a2 = -0.01)	theta = 5(a1 = 0a2 = -0.001)	theta = 15(a1 = 0a2 = -0.1)	theta = 15(a1 = -0.01a2 = -0.2)	theta = 15(a1 = -0.01a2 = -0.3)	theta = 15(a1 = 0a2 = -0.01)
a_1_	0.998	0.999	1.00	1.00	0.996	0.989	0.990	1.00
b_1_	0.965	0.984	0.989	0.997	0.995	0.970	0.941	0.990
**a2**	**0.981**	**0.991**	**0.988**	**0.984**	**0.967**	**0.999**	**0.998**	**0.991**
**b2**	**0.934**	**0.990**	**0.963**	**0.982**	**0.941**	**0.942**	**0.962**	**0.972**
a_3_	1.00	1.00	0.999	1.00	0.991	1.00	1.00	0.990
b_3_	1.00	1.00	1.00	1.00	1.00	1.00	1.00	1.00

**Table 14:** Power of test for nonlinear least square and nonlinear quantile fitting using error disttn as t(5) with a’s the coefficients of nonlinear model and b’s are the coefficients of quantile fit, with sample size equals 500.

	theta = 5(a1 = -0.01a2 = -0.1)	theta = 5(a1 = -0.01a2 = -0.001)	theta = 5(a1 = 0a2 = -0.01)	theta = 5(a1 = 0a2 = -0.001)	theta = 15(a1 = 0a2 = -0.1)	theta = 15(a1 = -0.01a2 = -0.2)	theta = 15(a1 = -0.01a2 = -0.3)	theta = 15(a1 = 0a2 = -0.01)
a_1_	1.00	1.00	1.00	1.00	1.00	1.00	1.00	1.00
b_1_	0.996	0.989	0.986	0.982	0.993	0.989	0.999	0.991
**a2**	**0.951**	**0.995**	**0.999**	**0.998**	**0.993**	**0.998**	**0.999**	**0.996**
**b2**	**0.960**	**0.990**	**1.00**	**0.997**	**0.999**	**1.00**	**0.999**	**1.00**
a_3_	1.00	1.00	1.00	1.00	1.00	1.00	1.00	1.00
b_3_	1.00	1.00	1.00	1.00	1.00	1.00	1.00	1.00

Additive outliers are included in the data before the comparison of the two above mentioned methods. We consider the following contaminated model with the addition of outliers

$$y_{t}^{c}=y_{t}+\vartheta \delta_{t}$$ (14)

Where $y_{t}^{c}$ denotes the response variable with contaminated data, *δ*_*t*_ = *y*_*t*_ + *ϑ* for some constant *ϑ* (theta used in the tables below) and *δ* equals 1 with probability “*π*” and “0” otherwise. This approach is also used by [26] and also by [27]. The difference here is that we are comparing it to the quantile fitting to the ESTAR model.

We fit the following model with the parameter values *a*'*s* are predefined for the nonlinear least square. We are interested in a_2_ less than zero, here we took a_3_ = 1 as in most economic applications of first order ESTAR

$$y_{t}=a_{1}+a_{3}\;*\;(y_{t-1}-a_{1})\;*\;\exp\{a_{2}\;*\;{\left(y_{t-1}-a_{1}\right)}^{2}\}+\varepsilon_{t}$$ (15)

and the following model with parameter values *b’s* are predefined for the nonlinear quantile fit

$$y_{t}=b_{1}+b_{3}\;*\;(y_{t-1}-b_{1})\;*\;\exp\{b_{2}\;*\;{\left(y_{t-1}-b_{1}\right)}^{2}\}+\varepsilon_{t}$$ (16)

The results are shown in the following Tables 10 to 14.

Tables 10 to 14 compares the power of the nonlinear least square fit and a type of robust fit called the quantile fit in the presence of outliers. In Tables 10 and 11 the error distribution in the data generation process is normal and in the other three tables the error is from t-distribution with 5-d.f. General quantile fit performs better in the presence of outliers because quantile fits take into account the median of the process instead of the mean and the median is not effected by outliers. We have computed the power of nonlinear time series ESTAR model to check the correct fitting of the nonlinear model when the data is generated from a nonlinear process. We can see from the above four tables that the performance of the quantile fit has improved in most of the situation especially when the contamination is high. The difference in the power performance of the two methods of model fitting remains unchanged for different sample sizes.

## Conclusion

We have tested unit root hypothesis against the ESTAR in the nonlinear framework and had obtained the critical values for testing the null hypothesis using non-normal errors (skewed and symmetric) for the Kapetanios test. We also have computed the size of the Kapetanios test for the sample size 50, 100, 250, 500 and 1000 and have compared it with the bootstrap testing procedure and found that the bootstrap procedure gives much closer results to the exact size of the test and hence reduces the problem of over sizing, which we have noticed in the simulations done by Cook [11]. The power of the wild bootstrap is computed and compared with the Kapetanios test statistics, which shows a significant improvement when wild bootstrap technique is used in case when the error is generated from normal distribution and Kapetanios test performs better in case when the error is generated from t-distribution. We have taken the error distribution in the DGP as normal in one case and t-disttn; with 5-degrees of freedom in the second case. In the last section we have estimated the power of ESTAR model on the basis of non-linear least square and have compared it with that of quantile fit which is supposed to be more robust and a type of median estimator. The R-code is defined for this simulations in S1 File. The results show for the data given in S2 File or S3 File, a slight increase in the power of the quantile fit for the nonlinear testing against the least square model fitting. Hence we conclude that the nonlinear quantile model fitting performs efficiently than the nonlinear least square model in the presence on outliers

## Supporting Information

S1 File R-Code file. (DOCX)

S2 File Data in.txt file. (TXT)

S3 File Data in.csv file. (CSV)
