# Supplementary material for: Unit Root Testing and Estimation in Nonlinear ESTAR Models with Normal and Non-Normal Errors
Source: PLoS One. 2016 Nov 29;11(11):e0166990. doi: 10.1371/journal.pone.0166990 (PMC5127548; doi:10.1371/journal.pone.0166990)
Supplement: S1 File — (DOCX) [file pone.0166990.s001.docx]

#**R-code**

################ model fitting with aditive outlier #################

S=1000

g=array( dim=c(S,4))

h=array( dim=c(S,4))

N <- 100; # sample size

phi <- 0.9; # ar coefficient of x process

ppi <- 0.1; # probability

zz <- 10; # contaminating process constant

inter <- 0.1;

arcoef1 <- 0.9;

arcoef2 <- -0.1;

gamma <- 1;

error <- rt(N,df=5);

x <- vector();

x[1:2] <- 0;

R=1;

error_nls=0;

while(R <= S && error_nls <= 500){

for(i in 3:N){

x[i] <- inter+(arcoef1*x[i-1]+arcoef2*x[i-2])*(1-exp(-gamma*(x[i-1]^2)))+

error[i];

}

#x <- filter(rnorm(N,0,1),phi,"rec");

delta <- sample(c(1,0),size=N,replace=T,prob=c(ppi,1-ppi));

z <- x+zz;

y <- x+z*delta;

ylag <- y[-N];

y <- y[-1];

nlmod <- try(nls(y~a1+((exp(a2*(ylag-a1)^2))*(ylag-a1)),start =list(a1=-.01,a2=.1),

control=list(maxiter=1000)),silent=T);

nqq <- try(nlrq(y~b1+((exp(b2*(ylag-b1)^2))*(ylag-b1)),start =list(b1=-.01,b2=.1),

control=list(maxiter=1000)),silent=T);

if(attributes(nlmod)[[1]][1]=="try-error" || attributes(nqq)[[1]][1]=="try-error"){

error_nls <- error_nls+1;

}else{

a=summary(nlmod)$coef

b=summary(nqq)$coef

g[R,]=a[,c(1,3)]

h[R,]=b[,c(1,3)]

R <- R+1;

}

}
